# A Threshold QuickDASH Score for Estimating a Diagnosis of Major Depression in Patients With Fingertip Injuries in the American and Dutch Population

**DOI:** 10.1177/15589447211060456

**Published:** 2021-12-30

**Authors:** Kamilcan Oflazoglu, Elfi M. Verheul, Taylor M. Pong, Marco J. F. P. Ritt, Hinne Rakhorst, Neal C. Chen

**Affiliations:** 1 Amsterdam UMC, Location VUmc, The Netherlands; 2 Medisch Spectrum Twente, Enschede, The Netherlands; 3 Harvard Medical School/Massachusetts General Hospital, Boston, MA, USA

**Keywords:** fingertip injury, major depression, upper extremity function

## Abstract

**Background:**

The aim was to determine the threshold Quick Disabilities of the Arm, Shoulder, and Hand (QuickDASH) score that estimates a diagnosis of major depression in patients with fingertip injuries in American and Dutch patients.

**Methods:**

In this observational cross-sectional study, 112 patients with a recent fingertip injury measured symptoms of depression with the Patient Health Questionnaire and upper extremity disability with the QuickDASH.

**Results:**

In the US cohort, 8 of 56 patients had an estimated diagnosis of major depression. A threshold value of QuickDASH of 50 showed a sensitivity of 88% and a specificity of 81%, with a negative predicting value (NPV) of 95% for an estimated diagnosis of major depression. In the Dutch cohort, 7 of 56 patients had an estimated diagnosis of major depression. The same threshold score of 50 had a sensitivity of 71%, a specificity of 63%, and an NPV of 94%.

**Conclusions:**

We have found a correlation between experienced loss of function and an estimated diagnosis of major depression in patients with a fingertip injury. Referral to the primary care physician for further evaluation of depression in these patients is advised.

## Introduction

Injuries of the fingertip are very common. Annually 4.8 million emergency department visits per year in the Unites States consist of crush, avulsion, laceration, and amputation injuries of the digits.^1^ In the Netherlands, there are 287 000 hand injuries each year, with the majority located in the finger.^2^

Given the importance of the hand function in daily living, injury to the fingertip can be limiting; however, the level of disability varies widely among patients with similar pathology. Some research gives us insights in that the magnitude of disability appears to correlate with the severity of depression in patients with hand illness.^3,4^ In fingertip injuries, it has been shown that symptoms of depression account for most of the variability in hand and arm-specific disability, pain intensity, and days to return to work.^5^ It is estimated that 1 in 8 patients with upper extremity conditions have major depression,^6^ and a Quick Disabilities of the Arm, Shoulder, and Hand (QuickDASH) score greater than 55 suggests a higher probability of an estimated diagnosis of major depression in patients with upper extremity illness.^7^

We wanted to extend this concept to a more homogeneous cohort of fingertip injuries. The primary aim of this study is to determine the threshold QuickDASH score that estimates a diagnosis of major depression in patients with fingertip injuries. The secondary aim of this study is to determine this threshold QuickDASH score in the Dutch population.

## Methods

### Study Design

This study was performed in an academic center in the United States and in a top-level trauma center in the Netherlands. The institutional review board of both centers approved this study and required that we excluded pregnant patients or patients not fluent in English or Dutch. All patients provided informed consent at enrollment.

### Power Analysis

Based on 2 previous studies that reported a prevalence of major depression of approximately 25% among orthopedic trauma patients,^3,8^ a sample size of 56 patients per center is needed to provide a power of 80% and an area under the curve (AUC) of 0.75, assuming a ratio of sample sizes of 3 between negative and positive groups.^7^

### Cohorts

In this observational study, adult patients with recent (within 7 days) fingertip injuries distal to the proximal interphalangeal joint who visited either the emergency department or the outpatient clinic were included. All consenting patients were asked to complete questionnaires to collect the following data: demographics (age, sex, race/ethnicity, dominant hand), injury-related demographics (level of injury and severity), history of depression, depression severity, and disability (upper extremity function).

Using a tablet computer, data were collected using REDCap (Research Electronic Data Capture) electronic data capture tools. The REDCap is a secure, Web-based software platform designed to support data capture for research studies, providing: (1) an intuitive interface for validated data capture; (2) audit trails for tracking data manipulation and export procedures; (3) automated export procedures for seamless data downloads to common statistical packages; and (4) procedures for data integration and interoperability with external sources.^9,10^

In the United States, the answers to all questions were completed in the presence of the researcher. In the Netherlands, only the demographics and injury-related questions were completed together with the researchers. When completed, patients received a link by e-mail to fill in the depression and disability questionnaires.

### Questionnaires

The 9-item Patient Health Questionnaire (PHQ-9) (Appendix) is a reliable instrument for measuring depression severity.^11^ It consists of questions that are based on the diagnostic criteria for depression according to the *Diagnostic and Statistical Manual of Mental Disorders*, fourth edition, and scored on a 4-point ordinal scale ranging from 0 (“not at all”) to 3 (“nearly every day”). The total score ranges from 0 to 27. A PHQ-9 score of 10 or greater has been suggested as a cutoff point for an estimated diagnosis of major depression to yield a high specificity and sensitivity.^11^

The QuickDASH is a validated 11-item questionnaire with 5-point Likert scale responses, which measures upper extremity–specific disability. The QuickDASH is widely used in hand surgery research. Test-retest reliability is 0.94, and Cronbach α is 0.90. The total score is scaled between 0 (no disability) and 100 (maximum symptoms and disability), with an average value of 10 among the general population of the United States.^12-14^

### Statistical Analysis

Variables were presented as frequencies and percentages for categorical variables and as mean with SD for continuous variables. In bivariate analysis, Fisher exact test was used for categorical variables and *t* test for QuickDASH score.

A receiver operating characteristic curve (ROC) was created to determine a threshold for QuickDASH score that corresponds with estimated diagnosis of major depression with a high sensitivity, specificity, and negative predicting value (NPV). In general (ie, ability to diagnose patients with and without the disease or condition based on the test), an AUC of 0.5 suggests no discrimination, 0.7 to 0.8 is considered acceptable, 0.8 to 0.9 is considered excellent, and more than 0.9 is considered outstanding.^15^

## Results

### Patient Characteristics

There were 80 men (71%) and 32 women (29%) in this study, with a mean age of 45 years (range, 20-82 years). Of the US cohort, only 2 patients declined participation; of the Dutch cohort, a total 20 patients did not complete both questionnaires after initial inclusion at the hospital and were excluded. Overall, in the 2 cohorts combined, most patients were white (88%) and did not smoke (87%). The dominant hand was affected in 61% of patients. In 50% (n = 56), there was an isolated distal phalangeal injury; in 18% (n = 20), there was a traumatic amputation; in 56% (n = 62), the phalanx was fractured; in 18% (n = 20), a crush injury occurred; in 63% (n = 70), fingers were lacerated; and in 29 fingers (26%), there was a nail avulsion. Half of all fingers were treated operatively. A history of depression was reported by 14% (n = 15) of the patients and a family history of depression by 11% (n = 12; Table 1).

**Table 1.** Characteristics of Patients With Fingertip Injury: Complete Cohort.

Characteristic	All patients
	(n = 112)
Age, mean (SD), y	45 (16)
Sex, n (%)	
Men	80 (71)
Women	32 (29)
Race, n (%)	
White	98 (88)
Other	14 (13)
Smoking, n (%)	
No	97 (87)
Yes	15 (13)
Dominant side affected, n (%)	
No	68 (61)
Yes	44 (39)
Level of injury, n (%)	
Middle phalanx	28 (25)
DIP joint	28 (25)
Distal phalanx	56 (50)
Amputation, n (%)	
No	92 (82)
Yes	20 (18)
Fracture, n (%)	
No	49 (44)
Yes	62 (56)
Crush, n (%)	
No	92 (82)
Yes	20 (18)
Laceration, n (%)	
No	42 (38)
Yes	70 (63)
Nail avulsion, n (%)	
No	83 (74)
Yes	29 (26)
Treatment, n (%)	
Conservative	55 (50)
Operative	55 (50)
Work status, n (%)	
Employed	70 (62)
Unemployed	42 (38)
History of depression, n (%)^a^	
No	97 (87)
Yes	15 (13)
Family history of depression, n (%)	
No	100 (89)
Yes	12 (11)
QuickDASH score, mean (SD)	41 (23)

*Note*. DIP = distal interphalangeal; QuickDASH = Quick Disabilities of the Arm, Shoulder, and Hand.

a All patients with a history of diagnosed depression have had treatment or were currently being treated for depression.

#### US cohort

Eight (14%) of 56 patients had an estimated diagnosis of major depression (PHQ-9 greater than 10). In bivariate analysis, unemployed patients had significantly higher rates of estimated diagnosis of major depression than employed patients, 46% versus 4.7%, respectively (*P* < .001). Furthermore, patients with a reported history of depression had significantly more often a PHQ-9 score of 10 or greater—8.9% of patients without a history of depression versus 36 % with a history of depression (*P* = .040; Table 2).

**Table 2.** Patient Characteristics and Factors Associated With an Estimated Diagnosis of Depression in Patients With Fingertip Injury: US Cohort.

Characteristic	All patients	Depression		*P* value
		No	Yes	
	(n = 56)	(n = 48)	(n = 8)	
Age, mean (SD), y	45 (13)	44 (14)	49 (8.6)	.28
Sex, n (%)				
Men	38 (68)	31 (65)	7 (88)	.41
Women	18 (32)	17 (35)	1 (13)	
Race, n (%)				.11
White	47 (84)	41 (85)	6 (75)	
Other	9 (16)	7 (15)	2 (25)	
Smoking, n (%)				
No	51 (91)	44 (92)	7 (88)	.55
Yes	5 (8.9)	4 (8.3)	1 (13)	
Dominant side affected, n (%)				> .99
No	22 (39)	19 (40)	3 (38)	
Yes	34 (61)	29 (60)	5 (63)	
Level of injury, n (%)				.46
Middle phalanx	9 (16)	8 (17)	1 (13)	
DIP joint	18 (32)	17 (35)	1 (13)	
Distal phalanx	29 (52)	23 (48)	6 (75)	
Amputation, n (%)				.37
No	43 (77)	38 (79)	5 (63)	
Yes	13 (23)	10 (21)	3 (38)	
Fracture, n (%)				.69
No	17 (31)	14 (30)	3 (38)	
Yes	38 (69)	33 (70)	5 (63)	
Crush, n(%)				.078
No	48 (86)	43 (90)	5 (63)	
Yes	8 (14)	5 (10)	3 (38)	
Laceration, n (%)				.45
No	31 (55)	28 (58)	3 (38)	
Yes	25 (45)	20 (42)	5 (63)	
Nail avulsion, n (%)				.24
No	34 (61)	31 (65)	3 (38)	
Yes	22 (39)	17 (35)	5 (63)	
Treatment, n (%)				.25
Conservative	33 (59)	30 (63)	3 (38)	
Operative	23 (41)	18 (38)	5 (63)	
Work status, n(%)				**< .001**
Employed	43 (77)	41 (85)	2 (25)	
Unemployed	13 (23)	7 (15)	6 (75)	
History of depression, n (%)				**.040**
No	45 (80)	41 (85)	4 (50)	
Yes	11 (20)	7 (15)	4 (50)	
Family history of depression, n (%)				.60
No	47 (84)	41 (85)	6 (75)	
Yes	9 (16)	7 (15)	2 (25)	
QuickDASH score, mean (SD)	34 (21)	31 (19)	57 (18)	**< .001**

*Note*. DIP = distal interphalangeal; QuickDASH = Quick Disabilities of the Arm, Shoulder, and Hand.

Values in bold are statistically significant values (*P* < .05).

### Threshold QuickDASH Score for Estimated Diagnosis of Major Depression (US)

**Figure 1.**
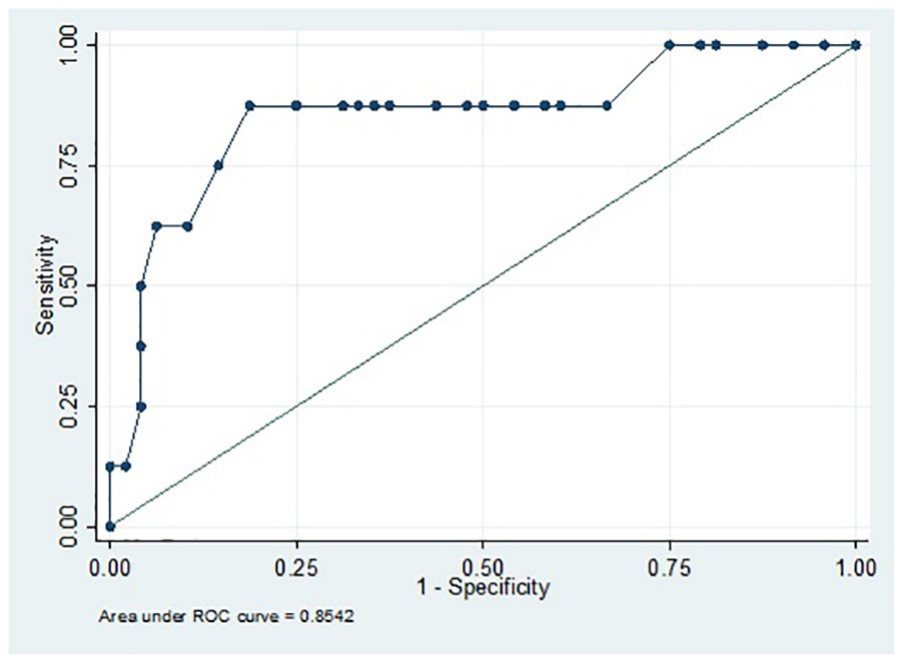
The area under the ROC curve of Quick Disabilities of the Arm, Shoulder, and Hand scores in patients with a fingertip injury for an estimated diagnosis of major depression in the US population. Area under the curve = 0.85; 95% confidence interval = 0.68-0.99. *Note.* ROC = receiver operating characteristic curve.

The mean QuickDASH score was 34 (SD = 21). Patients with an estimated diagnosis of major depression had a significantly higher QuickDASH score, mean of 57 (SD = 18) versus 31 (SD = 18; *P* < 0.001; Table 2). The AUC for QuickDASH score was 0.85 (95 % confidence interval [CI], 0.68-0.99) indicating that it is an excellent model for predicting an estimated diagnosis of major depression. A QuickDASH score of >50 had a sensitivity of 88% and a specificity of 81%, with an NPV of 95% (Table 3; Figure 1).

**Table 3.** ROC Analysis for Different Thresholds of QuickDASH Scores in Patients With a Fingertip Injury for Depression in the US Population.

Threshold, %	Sensitivity	Specificity
>0	100	0
>5	100	8.3
>10	100	13
>15	100	21
>20	88	33
>25	88	40
>30	88	52
>35	88	57
>40	88	66
>45	88	69
>50^a^	88	81
>55	63	90
>60	63	94
>65	50	96
>70	25	96
>75	13	100
>80	0	100

*Note.*^a^A threshold score of > 50 had a negative predictive value of 95%. QuickDASH = Quick Disabilities of the Arm, Shoulder, and Hand; ROC = receiver operating characteristic curve.

#### Dutch cohort

Seven (13%) of 56 patients had an estimated diagnosis of major depression in the Dutch cohort. In bivariate analysis, demographic variables, injury-related factors, and history of depression were not statistically different between patients with and without an estimated diagnosis of major depression (Table 4).

**Table 4.** Patient Characteristics and Factors Associated With an Estimated Diagnosis of Depression in Patients With Fingertip Injury: Dutch Cohort.

Characteristic	All patients	Depression		*P* value
		No	Yes	
	(n = 56)	(n = 49)	(n = 7)	
Age, mean (SD), y				
Sex, n (%)				.35
Men	42 (75)	38 (78)	4 (57)	
Women	14 (25)	11 (22)	3 (43)	
Race, n (%)				.50
White	51 (91)	45 (92)	6 (86)	
Other	5 (8.9)	4 (8.2)	1 (14)	
Smoking, n (%)				.60
No	46 (82)	41 (84)	5 (71)	
Yes	10 (18)	8 (16)	2 (29)	
Dominant side affected, n (%)				.22
No	36 (65)	33 (69)	3 (43)	
Yes	19 (35)	15 (31)	4 (57)	
Level of injury, n (%)				.076
Middle phalanx	27 (48)	26 (53)	1 (14)	
DIP joint	10 (18)	9 (18)	1 (14)	
Distal phalanx	19 (34)	14 (29)	5 (71)	
Amputation, n (%)				>.99
No	49 (88)	43 (88)	6 (86)	
Yes	7 (13)	6 (12)	1 (14)	.69
Fracture, n (%)				
No	32 (57)	27 (55)	5 (71)	
Yes	24 (43)	22 (45)	2 (29)	
Crush, n (%)				>.99
No	44 (78)	38 (78)	6 (86)	
Yes	12 (21)	11 (22)	1 (14)	
Laceration, n (%)				.32
No	11 (20)	11 (22)	0	
Yes	45 (80)	38 (78)	7 (100)	
Nail avulsion, n (%)				.21
No	49 (88)	44 (90)	5 (71)	
Yes	7 (13)	5 (10)	2 (29)	
Treatment, n				.22
Conservative	24 (43)	23 (47)	1 (14)	
Operative	32 (57)	26 (53)	6 (86)	
Work status, n (%)				.42
Employed	27 (48)	25 (51)	2 (29)	
Unemployed	29 (52)	24 (49)	5 (71)	
History of depression, n (%)				.072
No	52 (93)	47 (96)	5 (71)	
Yes	4 (7.1)	2 (4.1)	2 (29)	
Family history of depression, n (%)				>.99
No	53 (95)	46 (94)	7 (100)	
Yes	3 (5.4)	3 (6.1)	0	
QuickDASH score, mean (SD)	47 (23)	44 (22)	69 (17)	**<.001**

*Note*. DIP = distal interphalangeal; QuickDASH = Quick Disabilities of the Arm, Shoulder, and Hand.

Values in bold are statistically significant values (*P* < .05).

### Threshold QuickDASH Score for Estimated Diagnosis of Major Depression (Dutch)

**Figure 2.**
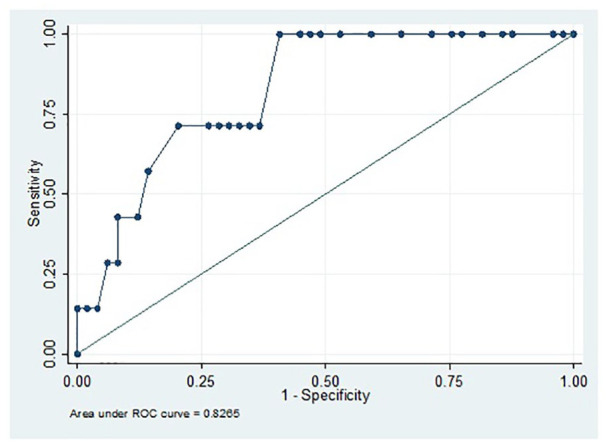
The area under the ROC curve of Quick Disabilities of the Arm, Shoulder, and Hand scores in patients with a fingertip injury for an estimated diagnosis of major depression in the Dutch population. Area under the curve = 0.83; 95% of confidence interval = 0.69-0.99. *Note*. ROC = receiver operating characteristic curve.

The mean QuickDASH score was 47 (SD = 22). Similar to the US cohort, patients with an estimated diagnosis of major depression had a significantly higher QuickDASH score, mean of 69 (SD = 17) versus 44 (SD = 22; *P* < .001; Table 4). The AUC for QuickDASH score was 0.83 (95 % CI, 0.69-0.99). A QuickDASH score of >50 had a sensitivity of 71% and a specificity of 63% with an NPV of 94%, lower than in the US cohort (Table 5; Figure 2).

**Table 5.** ROC Analysis for Different Thresholds of QuickDASH Scores in Patients With a Fingertip Injury for Depression in the Dutch Population.

Threshold, %	Sensitivity	Specificity
>0	100	0
>5	100	0
>10	100	2
>15	100	4.1
>20	100	14
>25	100	22
>30	100	29
>35	100	34
>40	100	51
>45	100	55
>50^a^	71	63
>55	71	67
>60	71	71
>65	71	75
>70	57	86
>75	42	92
>80	29	94

*Note.*^a^A threshold score of > 50 had a negative predictive value of 94%. QuickDASH = Quick Disabilities of the Arm, Shoulder, and Hand; ROC = receiver operating characteristic curve.

## Discussion

Early diagnosis of depression is important because it can shorten the disease period, reduce negative thoughts, increase compliance for therapy, and lower the experienced loss of function.^4,16^ Loss of function in one of the upper extremities can result in major depression.^4,6^ The aim was to determine a threshold value of the QuickDASH score to predict the risk of estimated diagnosis of major depression in patients with a fingertip injury in the US and Dutch population. The prevalence of estimated diagnosis of major depression was 14% in the US cohort and 13% in the Dutch cohort. We found that a threshold value of QuickDASH of 50 showed a sensitivity of 88% and a specificity of 81%, with an NPV of 95% in the US population. The same threshold score of 50 had a sensitivity of 71%, a specificity of 63%, and an NPV of 94% in the Dutch population. History of depression and history of unemployment were significantly associated with estimated diagnosis of major depression in the US population.

The prevalence of depression of 13% to 14% in this study is in line with a previous study about depression in patients with upper extremity conditions.^6^ The prevalence of depression of orthopedic trauma inpatients was 20% using the PHQ-9.^17^ The prevalence of depression in the Netherlands is 7.4% in the general population, with a 12-month prevalence of 5.2%.^18^ No data are available of the PHQ-9 tested on the general Dutch population. Similar to patients with upper extremity conditions, personal history of depression was significantly associated with an estimated diagnosis of major depression in patients with a fingertip injury.^6^

We found a significantly higher QuickDASH score in patients with an estimated major depression. These results are in line with Molleman et al.^7^ The study of Overbeek et al showed a moderate correlation between the QuickDASH and the Patient-Reported Outcomes Measurement Information System of depression. Both studies support the relationship between the severity of the disability and the outcome of depression.^19^

In our study, a threshold QuickDASH score of 50 had a sensitivity of 88%, a specificity of 81%, and an NPV of 95% in the US population. With an NPV of 95%, we can conclude that just 1 in 20 patients with a fingertip injury with a QuickDASH score of less than 50 will have risk of major depression, Furthermore, almost 9 in 10 patients with major depression will have a QuickDASH score of 50 or greater. Molleman et al^7^ found that a DASH score of 55 or greater in patients with common upper extremity disorders had a predictive value for an estimated diagnosis of major depression.

In the US cohort, unemployed patients were more likely to have estimated diagnosis of major depression compared with employed patients. This is supported by the study of Nurmela et al,^20^ which showed that a longer duration of unemployment results in more people suffering from major depression. This is an indirect association caused by the adverse effects of unemployment, such as financial lack, social distancing, and less self-esteem.^21^ Cheng et al^22^ found an association between employment status and depression and showed a correlation to age. In contrast to our results, Wittayanukorn et al^23^ found that sex and race were also associated with depression.

There are limitations to our study. First, although we tried to include as many patients as possible during their initial presentation at the emergency department, some patients might be missed because of patient flows in that department. It is unclear how many patients were missed. Second, this study is based on 2 questionnaires that target different time frames. The PHQ-9 questionnaire asks for symptoms of the last 2 weeks and the QuickDASH for the last week. All patients were aware of their injury when filling in both questionnaires and estimated their inability as good as possible. However, we do not believe this led to different outcomes of PHQ-9 or QuickDASH scores. Although a PHQ-9 score of 10 or greater had a specificity of 88% and a sensitivity of 88% for major Depression,^11,24,25^ it should still be diagnosed through a formal psychiatric evaluation. Third, 20 (26%) of 76 consenting patients in the Dutch cohort did not complete the PHQ-9 and QuickDASH questionnaires after they were included in the study and agreed to complete the questionnaires through a link by e-mail. Fourth, as this is a cross-sectional study, all statistical analyses in this study are performed on the baseline measurements. Fifth, not all hand surgeons use the QuickDASH; however, the goal is to see whether QuickDASH as a surrogate of function has meaningful associations with other problems, such as depression. Regardless of whether a surgeon uses QuickDASH or not, when a patient expresses poor function after these injuries, it may be worthwhile being aware that poor function is multifactorial and may not limited to just hand dysfunction. Finally, our findings are statistical associations and do not establish causality. However, regarding treatment, in some ways it is irrelevant whether the depression is caused by the injury or vice versa as patients should be treated for both their fingertip injury and depression, if present. Finally, more than half of Dutch patients were unemployed. This could be explained as inclusion of the Dutch patients happened mostly during the COVID pandemic, in which many individuals were not able to work, whereas the inclusion of the US population was prior to the COVID pandemic.

The impact of a relatively small injury like a fingertip injury could have major impact on one’s life and should, therefore, not be underestimated. We have found a correlation between experienced loss of function and an estimated diagnosis of major depression in patients with a fingertip injury. Referral to the primary care physician, therapist, or medical social worker for further evaluation of depression in these patients is advised. We recognize that it poses a challenge for unemployed patients, especially in the United States. Early treatment of both the injury and depression will probably lead to a better compliance for (hand) therapy and therefore to better overall results. Finally, this study improved the research infrastructure between Dutch and American centers. We aim to have more collaborative studies between nations.

## Appendix

### Patient Health Questionnaire (PHQ-9)

Over the last 2 weeks, how often have you been bothered by any of the following problems?

Little interest or pleasure in doing things

- Not at all
- Several days
- More than half the days
- Nearly every day

Feeling down, depressed, or hopeless

- Not at all
- Several days
- More than half the days
- Nearly every day

Trouble falling or staying asleep, or sleeping too much

- Not at all
- Several days
- More than half the days
- Nearly every day

Feeling tired or having little energy

- Not at all
- Several days
- More than half the days
- Nearly every day

Poor appetite or overeating

- Not at all
- Several days
- More than half the days
- Nearly every day

Feeling bad about yourself—or that you are a failure or have let yourself or your family down

- Not at all
- Several days
- More than half the days
- Nearly every day

Trouble concentrating on things, such as reading the newspaper or watching television

- Not at all
- Several days
- More than half the days
- Nearly every day

Moving or speaking so slowly that other people could have noticed? Or the opposite—being so fidgety or restless that you have been moving around a lot more than usual

- Not at all
- Several days
- More than half the days
- Nearly every day

Thoughts that you would be better off dead or of hurting yourself in some way

- Not at all
- Several days
- More than half the days
- Nearly every day
